# *In vivo* manipulation of the protein homeostasis network in rhabdomyosarcoma

**DOI:** 10.18632/oncotarget.28764

**Published:** 2025-08-29

**Authors:** Kristen Kwong, Yue Pan, Jacqueline Morales, Matthew Watson, David V. Allegakoen, Alex G. Lee, Trever G. Bivona, Peter Wipf, Christopher J. Guerriero, Jeffrey L. Brodsky, Amit J. Sabnis

**Affiliations:** ^1^Department of Pediatrics, Division of Oncology, University of California San Francisco, San Francisco, CA 94143, USA; ^2^Department of Pediatrics, Georgetown University, Washington, DC 20057, USA; ^3^Department of Medicine, Division of Hematology-Oncology, University of California San Francisco, San Francisco, CA 94143, USA; ^4^Chan Zuckerberg Biohub, San Francisco, CA 94158, USA; ^5^Department of Chemistry, University of Pittsburgh, Pittsburgh, PA 15260, USA; ^6^Department of Biological Sciences, University of Pittsburgh, Pittsburgh, PA 15260, USA

**Keywords:** protein homeostasis, rhabdomyosarcoma, unfolded protein response, preclinical therapeutics, p97

## Abstract

The protein homeostasis (proteostasis) network includes quality control systems that coordinate protein synthesis, folding, localization, and degradation, and is deregulated in numerous diseases including cancer. Loss of proteostasis can activate lethal cellular stress responses, potentially opening a therapeutic window. Previous research demonstrated that MAL3-101, an inhibitor of heat shock protein 70-kD (HSP70) chaperones, selectively induces rhabdomyosarcoma (RMS) cell death via unfolded protein response (UPR) activation. RMS is the most common pediatric soft tissue sarcoma, and relapsed patients are rarely cured despite transient responses to DNA-damaging therapy. To examine whether MAL3-101 or more drug-like proteostasis inhibitors represent a new therapeutic strategy for RMS, we screened proteostasis components that might recapitulate the effects of MAL3-101 *in vivo*. We find that inhibition of *VCP*, which encodes the p97 ATPase that facilitates proteasome-dependent degradation, similarly activates the UPR and induces RMS apoptosis. In mouse models, a preclinical p97 inhibitor showed superior bioavailability and anti-tumor activity compared to MAL3-101. Patient-derived xenografts exhibited a spectrum of p97 inhibitor sensitivities, and RNA sequencing of resistant tumors revealed elevated autophagy, nominating a biomarker of proteostasis adaptability. Together, these findings confirm that proteostasis inhibition can slow RMS growth and suggest that targeting compensatory network components might yield synergistic outcomes.

## INTRODUCTION

Rhabdomyosarcoma (RMS) is a mesenchymal cancer that affects children and young adults. Intensification of treatment with genotoxic modalities, including multi-agent chemotherapy and ionizing radiation, have not significantly improved outcomes for patients with high risk clinical features which include metastatic disease, the presence of *FOXO1* fusions, and older age at presentation [1, 2]. Unfortunately, then, related therapies that elicit an apoptotic DNA damage response are unlikely to meaningfully change the dismal five-year survival of <20% in this patient subgroup.

Imbalances in the cellular machinery that oversees protein synthesis, folding, transport, and degradation, which has been termed proteostasis, result in cellular stresses that activate apoptotic signaling pathways independent of DNA damage [3]. Therefore, we and others previously proposed that increased demands on protein synthesis, protein imbalance arising from aneuploidy, and oxidative stress in cancer cells generally [4–8]—and in RMS in particular [9]—might lower the threshold for proteotoxic cell death. Indeed, we previously reported that genetic loss or chemical inhibition of the cytosolic heat shock protein 70 (HSP70) provokes a lethal unfolded protein response (UPR) in RMS cells [6, 10]. The UPR is a coordinated stress response pathway that permits either adaptation to an accumulation of misfolded proteins in the endoplasmic reticulum (ER), or commits cells to apoptotic death in the face of this irreparably deranged protein folding compartment [11]. Consistent with these data, we showed that RMS cells treated with HSP70 inhibitors activated the PERK-eIF2a-CHOP arm of the UPR, culminating in cancer cell apoptosis [6, 10].

Our prior findings support the broader hypothesis that chemical manipulation of the proteostasis network, using preclinical compounds, might similarly activate stress responses to trigger apoptosis *in vivo*, thereby uncovering new strategies to elicit RMS-selective cell death. Here, we use transcriptional profiling to nominate a cellular AAA-ATPase, p97, which is encoded by *VCP* and is required to support myriad cellular activities, including proteasome-dependent degradation of non-native proteins [12, 13], as a pharmacologically tractable target in RMS. Consistent with these data and the generation of *in vivo* compatible p97-targeting drugs [14], we used xenograft models both to describe the efficacy of VCP inhibition in RMS disease models and to dissect potential compensatory mechanisms that limit response or induce resistance. Our results support the hypothesis that the proteostasis network plays a critical role in RMS cell survival and that effective therapeutic targeting of distinct factors in this network will also require inhibition of compensatory proteostasis pathway components.

## RESULTS

### Inhibition of p97 recapitulates the downstream effects of HSP70 inhibition in RMS

The human genome encodes 14 HSP70 isoforms which promote a variety of cellular processes, including protein folding, preventing protein aggregation, and regulating protein assembly and trafficking [15]. The small molecule MAL3-101 directly binds and inhibits HSP70 activity by preventing the acceleration of the chaperone’s rate of ATP hydrolysis in the presence of J-domain protein co-chaperones [16–18]. As the effect of J-domain protein interaction is conserved across all HSP70 isoforms [19], MAL3-101 could, in theory, exert its effects on cells through inhibition of multiple targets, making it difficult to reproduce its effects pharmacologically.

**Figure 1:**
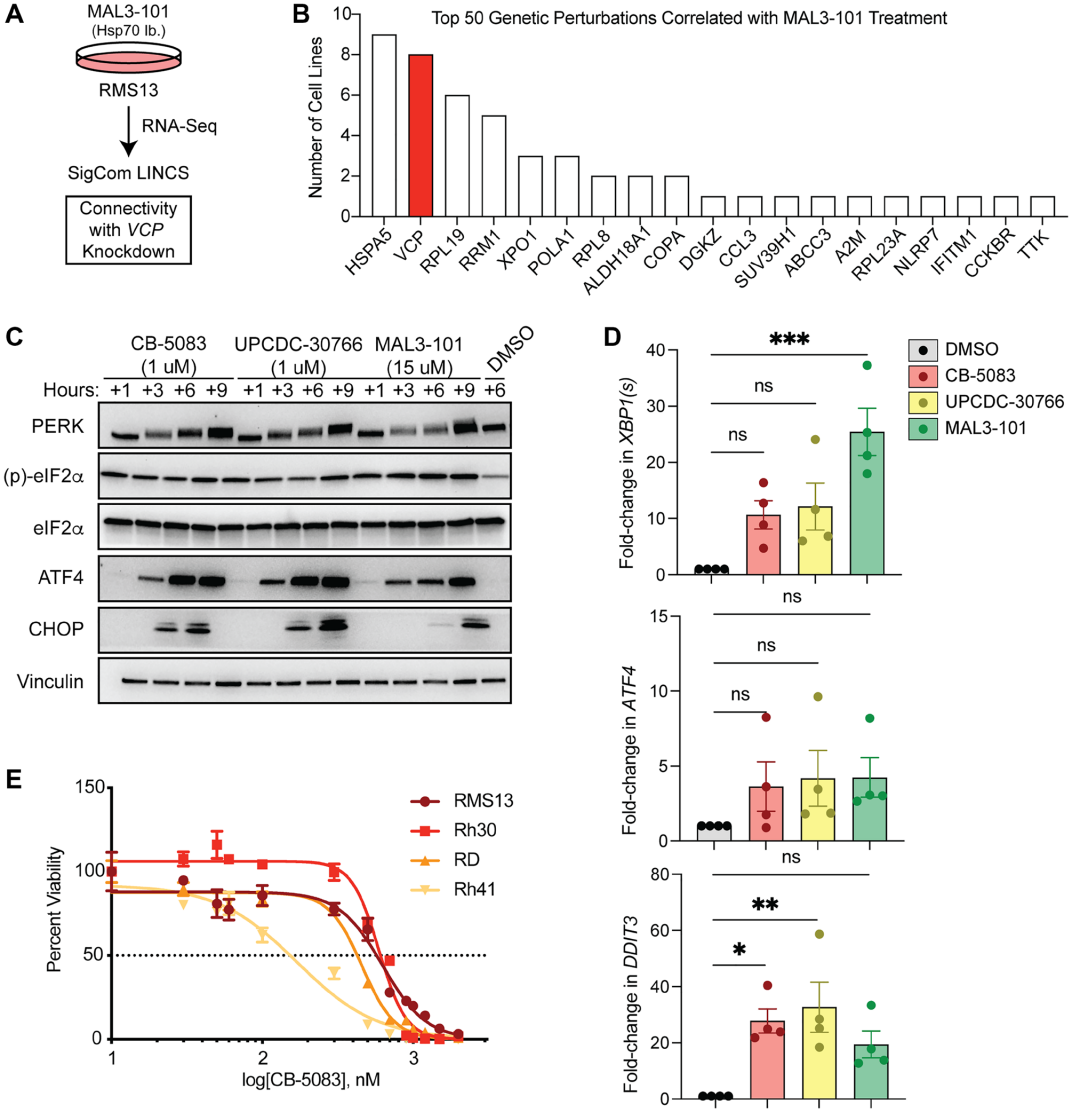
p97 inhibition activates the unfolded protein response and induces apoptosis in rhabdomyosarcoma. (**A**) RMS13 cells were treated with DMSO or 10 micromolar MAL3-101 for 9 hours, and total RNA was extracted and subjected to whole transcriptome sequencing. Differentially expressed genes (log2 fold-change ≥1 or ≤−1), treated with MAL3-101 compared to DMSO were uploaded to SigCom LINCS to identify genetic perturbations in a panel of cell lines with matching transcriptional effects. (**B**) The top 50 such genetic perturbations showed that loss of *VCP*, encoding the ATPase p97, resulted in similar transcriptomes in 8 cell lines. (**C**) Immunoblots from RMS13 cells treated with the p97 inhibitors CB-5083 and UPCDC-30766 or the HSP70 inhibitor MAL3-101 show similar kinetics of UPR activation. Phosphorylated, active PERK demonstrates a slower migration and upward shift. (**D**) The effects of these compounds on the transcription of UPR targets, as measured by qPCR for spliced *XBP1* (*XBP1(s)), ATF4,* and *DDIT3* (*p*-value by one-way ANOVA = 0.0015, 0.3551, and 0.0076 respectively; *p*-values by Dunnett’s post-hoc multiple comparisons test are shown, ^*^ *p* < 0.05, ^**^ *p* < 0.01, ^***^ *p* < 0.001, Abbreviation: ns: not significant). (**E**) Four patient-derived rhabdomyosarcoma cell lines were treated with increasing doses of CB-5083 and viability analyzed by CellTiterGlo. EC_50_ doses are in the sub-micromolar range.

To identify other proteostasis network components that might be targeted more specifically with drug-like compounds, we considered the top deregulated genes from RNASeq of MAL3-101-treated RMS13 cells [10] and compared them to single-gene CRISPR perturbations in other cell lines using the SigCom LINCS platform [20, 21] (Figure 1A, 1B and Table 1). The most common gene whose loss matched the transcriptional effects of MAL3-101 in RMS cells was the ER-resident HSP70, *HSPA5*, which is more commonly known as GRP78 or BiP. GRP78 is essential to maintain ER proteostasis by virtue of its ability to support the import, folding, and modification of proteins in the ER, as well as for the degradation of misfolded proteins in the ER [22]. *HSPA5* is also a major target of the UPR [23, 24]. Thus, these data are in line with a MAL3-101-dependent perturbation of ER proteostasis.

**Table 1:** Top correlations with MAL3-101 treatment by Z-score in LINCS L1000 CRISPR perturbations

Gene	Name	Z-score	*p*-value (Bonferroni)
*VCP*	Valosin containing protein/p97	14.081	6.95E-319
*RPL19*	Ribosomal protein L19	13.809	6.95E-319
*DGKZ*	Diacylglycerol kinase zeta	13.162	6.95E-319
*HSPA5*	Hsp70 member 5/GRP78/BiP	12.979	6.95E-319
*CCL3*	C-C Motif chemokine ligand 3	12.844	6.95E-319
*XPO1*	Exportin 1	12.266	6.95E-319
*SUV39H1*	SUV39H1 histone lysine methyltransferase	12.221	6.95E-319
*RRM1*	Ribonucleotide reductase catalytic subunit M1	12.157	6.95E-319
*ABCC3*	ATP binding cassette subfamily C member 3	12.010	6.95E-319
*A2M*	Alpha-2 microglobulin	11.955	6.95E-319

Interestingly, the gene whose knockdown had the highest Z-score was *VCP* (Table 1), which encodes the p97 AAA ATPase. As noted above (and see, e.g., [12, 13]), p97 functions as an energy-requiring modifier of protein function [25]. As a proteostasis component, p97 powers the dislocation of misfolded proteins from the ER to the proteasome in a process known as ER-associated degradation (ERAD) [26], and is also required for induction and maintenance of the autophagy pathway [27]. It is also noteworthy that autophagy provides a second route to degrade proteins in the cytosol, mitochondria, as well as in the ER [28, 29]. As anticipated, CRISPR-mediated knockout (KO) of the *VCP* gene phenocopied the transcriptional effects of MAL3-101 treatment in multiple cell lines in the LINCS database (Figure 1B). Knockdown of *VCP* by shRNA in RMS cells showed similar effects on the activation of several UPR targets and decreased cellular fitness in growth assays (Supplementary Figure 1A–1D, and also see below). In addition, knockout of the gene encoding *VCP* slows the growth of multiple cancer cell lines, including RMS cell lines, as represented in the Dependency Map project (Supplementary Figure 2), a comprehensive CRISPR/Cas9-based analysis of pan-cancer genetic dependencies [30]. Therefore, *VCP*/p97 might represent a more potent target for therapy than *HSPA8*/HSP70.

To test this hypothesis, we inhibited p97 with CB-5083, a small molecule inhibitor tested in phase 1 studies for various cancers [31, 32]. Clinical trials with this ATP-competitive compound were ultimately stopped due to its inhibition of PDE6 and corresponding off-target effects on the visual system [33]. Yet, new p97 inhibitors are continuously being developed [34], and a CB-5083 analog with lessened off-target effects entered clinical trials and showed superior effects in various models [14, 35]. Consequently, CB-5083 represents an immediately available tool compound to test our hypothesis that *in vivo* disruption of proteostasis, independent of HSP70 inhibition, is a therapeutically tractable strategy in RMS. We also used a structurally distinct inhibitor, UPCDC-30766, to confirm the on-target effects of p97 inhibition. While CB-5083 is an ATP-binding site inhibitor [31], the 1,2,4-triazole UPCDC-30766 is an allosteric site modulator of p97 [36]. Therefore, we reasoned that shared transcriptional and biochemical effects of these agents would confirm the on-target activities of p97 inhibition and offer a path to study additional p97 inhibitors as they are developed.

To this end, we treated RMS cell lines with CB-5083, UPCDC-30766, or MAL3-101 and measured activation of the UPR as a biomarker of proteostasis. All three drugs increased eIF2α phosphorylation and protein levels of ATF4 and CHOP, which are respectively an ER stress-responsive transcription factor and a pro-apoptotic transcription factor (Figure 1C). In addition, we observed robust induction of the spliced *XBP1* transcript (a marker of the IRE1-mediated arm of the UPR), and transcriptional upregulation of *ATF4* along with *DDIT3*, which encodes CHOP (Figure 1D). Moreover, both CB-5083 and UPCDC-30766 were effective at lower doses compared to MAL3-101 (Figure 1C). In cell proliferation assays, CB-5083 and UPCDC-30766 killed RMS cell lines at sub-micromolar IC_50_ (Figure 1E and Supplementary Figure 1E). These doses of CB-5083 are similar to those seen in efficacy studies in multiple myeloma cell lines [37], a cancer model in which drugs that target various nodes in the proteostasis network have entered the clinic and are undergoing further development [38].

### Proteostasis inhibitor efficacy correlates with pharmacokinetic exposure in xenograft models

**Figure 2:**
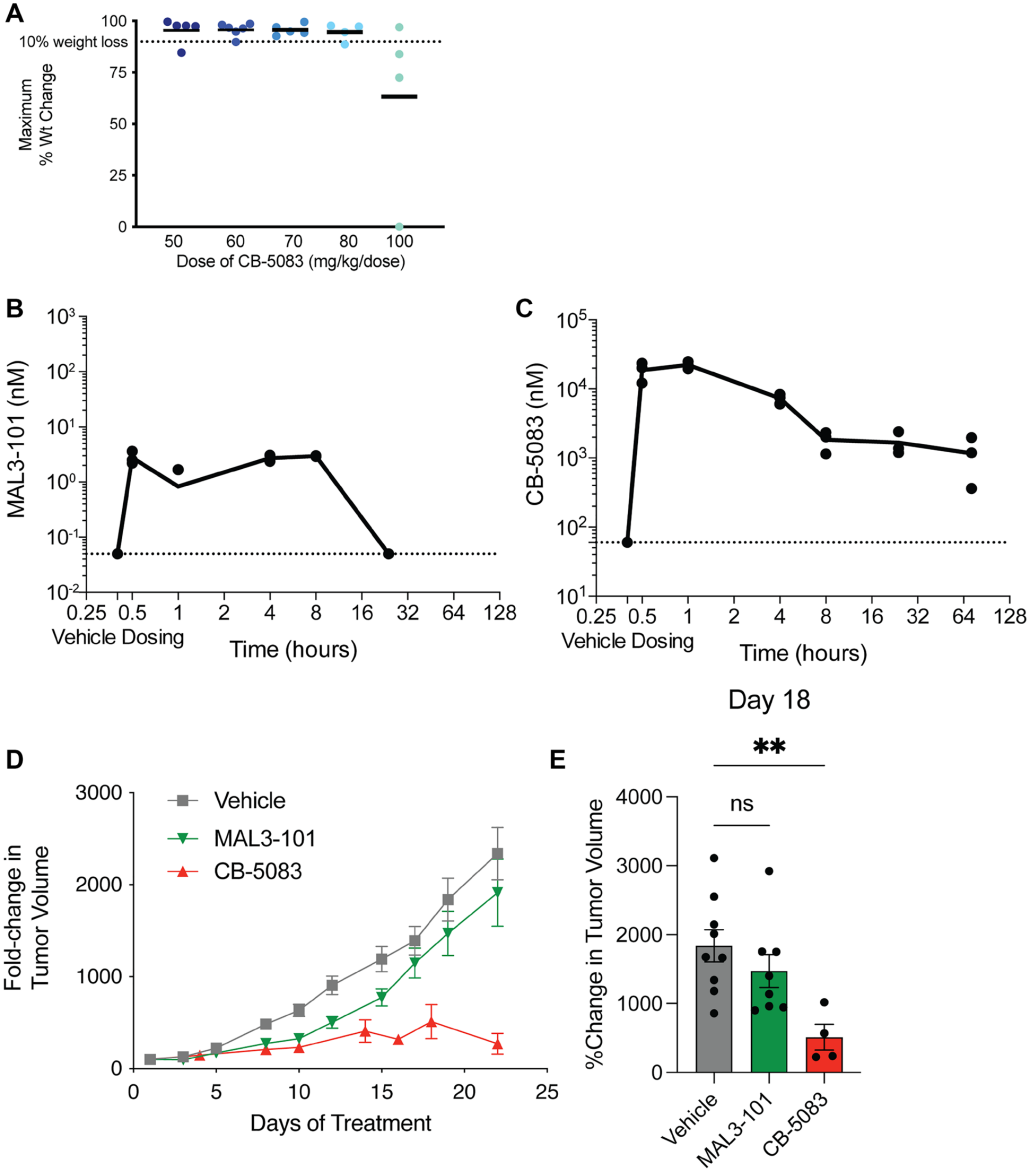
CB-5083 is more potent than MAL3-101 in RMS cell line xenografts. (**A**) *nu/nu* mice were treated with CB-5083 by oral gavage for four continuous days at the indicated doses and weight was measured on day 8. 80 mg/kg was established as the maximum tolerated dose (MTD) in this strain. Mice were treated with the MTD of (**B**), MAL3-101 (50 mg/kg IP injection) or (**C**) CB-5083 (80 mg/kg OG) and euthanized at the indicated timepoints. Cardiac blood was sampled and submitted for drug quantification by LC/MS-MS. Cmax for MAL3-101 was below its *in vitro* EC_50_, but not for CB-5083. (**D**) *nu/nu* mice were implanted with 1 × 10^6^ RMS13 cells in MatriGel and began treatment once tumors were >10 mm in maximum diameter. MAL3-101 slowed initial tumor growth, whereas CB-5083 had a more sustained effect. (**E**) tumor volumes after 21 days of treatment; mean tumor volume compared with either drug treatment to vehicle using a two-sided student’s *t*-test, ^**^ *p* < 0.01.

We next asked whether the *in vitro* sensitivities of RMS cell lines to MAL3-101 and CB-5083 would extend to an *in vivo* xenograft model. Mice treated with MAL3-101 at 55 mg/kg every other day did not show any substantial weight loss or toxicity, whereas higher doses or daily dosing led to a moribund condition within 24 hours, which was absent in mice receiving vehicle alone (data not shown). In contrast, CB-5083 at up to 80 mg/kg was tolerated in *nu/nu* mice without significant weight loss after one week (Figure 2A). Single doses comparing these two agents also showed that MAL3-101 had prolonged retention time in plasma, but had a peak concentration below the *in vitro* IC_50_ of 2.6 μM (Figure 2B). By contrast, CB-5083 achieved plasma concentrations above the *in vitro* IC_50_ of 650 nM after a single dose delivered by oral gavage and was detected after three days (Figure 2C). These differences in pharmacokinetic properties translated readily into distinct efficacies in xenografts (Figure 2D, 2E). While MAL3-101 initially slowed tumor growth, after three weeks this effect was no longer significant. CB-5083 instead offered prolonged inhibition of tumor growth relative to the vehicle control.

### RMS patient-derived xenografts show divergent response to CB-5083

**Figure 3:**
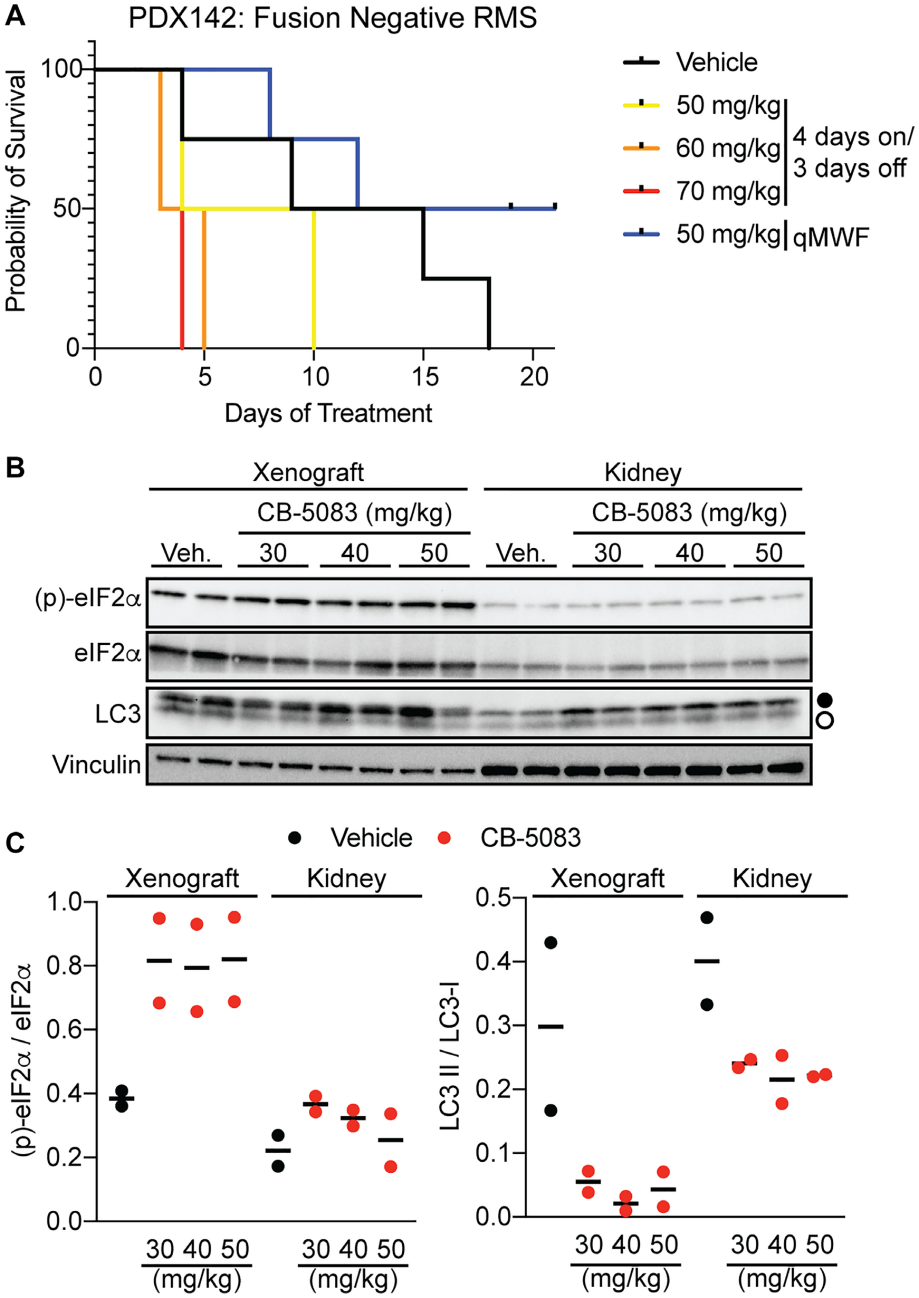
CB-5083 shows dose-dependent toxicity and UPR activation in RMS patient-derived xenografts. (**A**) NSG mice were implanted with the anaplastic rhabdomyosarcoma patient-derived xenograft 142 and treated with the indicated doses of CB-5083 either 4 days on/3 days off (as with *nu/nu* mice) or every other day. Early lethality was seen with continuous dosing in the NSG model, but could be rescued by interrupted dosing at 50 mg/kg. (**B**) mice treated with a single indicated dose of CB-5083 were euthanized after 24 hours and xenografts and kidneys harvested for immunoblots. Closed circle, LC3-I; open circle, LC3-II. Results are quantified in (**C**) phosphorylation of eIF2α is seen at the NSG MTD of 50 mg/kg in tumor xenografts, though minimally in kidney. Inhibition of autophagy (accumulation of LC3-I) is seen in both tissues.

Based on the results in cell line xenografts, we next studied the effects of CB-5083 on RMS tumor growth *in vivo* using patient-derived xenografts (PDX), which reflect more of the diverse underlying genomic drivers of RMS [39]. We selected a fusion negative RMS model and three *FOXO1* fusion positive models (one *PAX3-FOXO1*, two *PAX7-FOXO1*) (Table 2). NSG mice required an interrupted dosing schedule (three days a week) with a much lower MTD of 50 mg/kg (Figure 3A and data not shown) than *nu/nu* mice. Nonetheless, the UPR was still induced in isolated xenografts from the PDX models, as indicated by significantly increased phosphorylation of eIF2α (Figure 3B, 3C), which slows global translation and can also lead to CHOP induction [40]. As a control, we then measured whether the UPR was induced in the kidney, which was selected based on the known sensitivity of this organ to UPR-inducing drugs and links between the UPR and renal disease [41]. However, the levels of p-eIF2α were unchanged compared to the vehicle control. Interestingly, there was an accumulation of LC3-I in both tissues (Figure 3B, 3C, filled circles), which suggests that CB-5083 acutely inhibits autophagy. This outcome is consistent with the established role of p97 in autophagy [27]. Together, these results suggest the existence of a threshold effect for UPR activation within RMS xenografts.

**Table 2:** Patient-derived xenografts used in this study

PDX ID	Driver	Age	Source	Full name
PDX142	*TP53* p.R282W	7 yo	UCSF	
PDX640	*PAX7-FOXO1*	16 yo	UCSF	
PDX759	*PAX3-FOXO1*	19 yo	St Jude CSTN	SJRHB013759_X1
PDX156	*PAX7-FOXO1, FGFR4* p.V550E	16 yo	St Jude CSTN	SJRHB046156_X1

**Figure 4:**
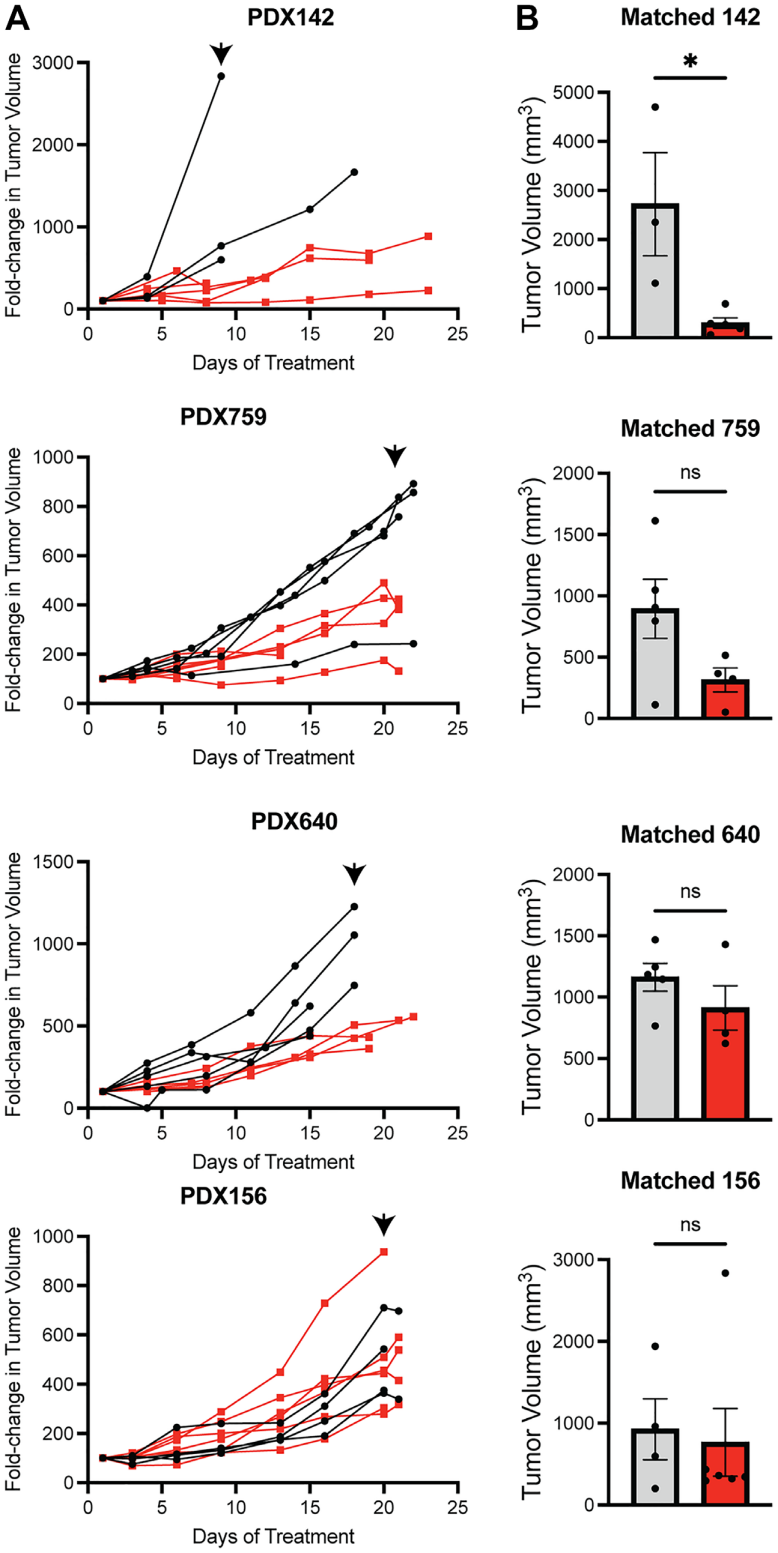
RMS PDX show a range of intrinsic resistance to CB-5083. (**A**) fold change in tumor volumes is plotted for four different RMS PDX models (black, vehicle; red, CB-5083 50 mg/kg OG every Monday, Wednesday, and Friday). Animals were euthanized when maximum tumor diameter exceeded 2 cm. (**B**) Bar graphs compare mean tumor volumes at the latest treatment point when >2 vehicle-treated mice were surviving (indicated by arrowheads in A) using a two-sided student’s *t*-test. ^*^ *p* < 0.05.

Based on these data, we subsequently treated mice harboring the four PDX models for 3 weeks with CB-5083 and uncovered a spectrum of responses (Figure 4A, 4B). CB-5083 produced a statistically significant decrease in tumor volumes in the PDX142 (fusion negative) model. In the PDX759 (PAX3-FOXO1) model, slowed tumor growth throughout the treatment regimen was evident without reaching statistical significance at the study endpoint. Interestingly, there was little effect after CB-5083 treatment in the PDX640 model and no effect in the PDX156 line, both of which are PAX7-FOXO1 models. These data indicate that inhibition of the p97 arm of the proteostasis network will have to be combined with other therapies for a meaningful anti-tumor effect (see below and Discussion).

### Distinct responses to CB-5083 are evident in sensitive and resistant PDX models

**Figure 5:**
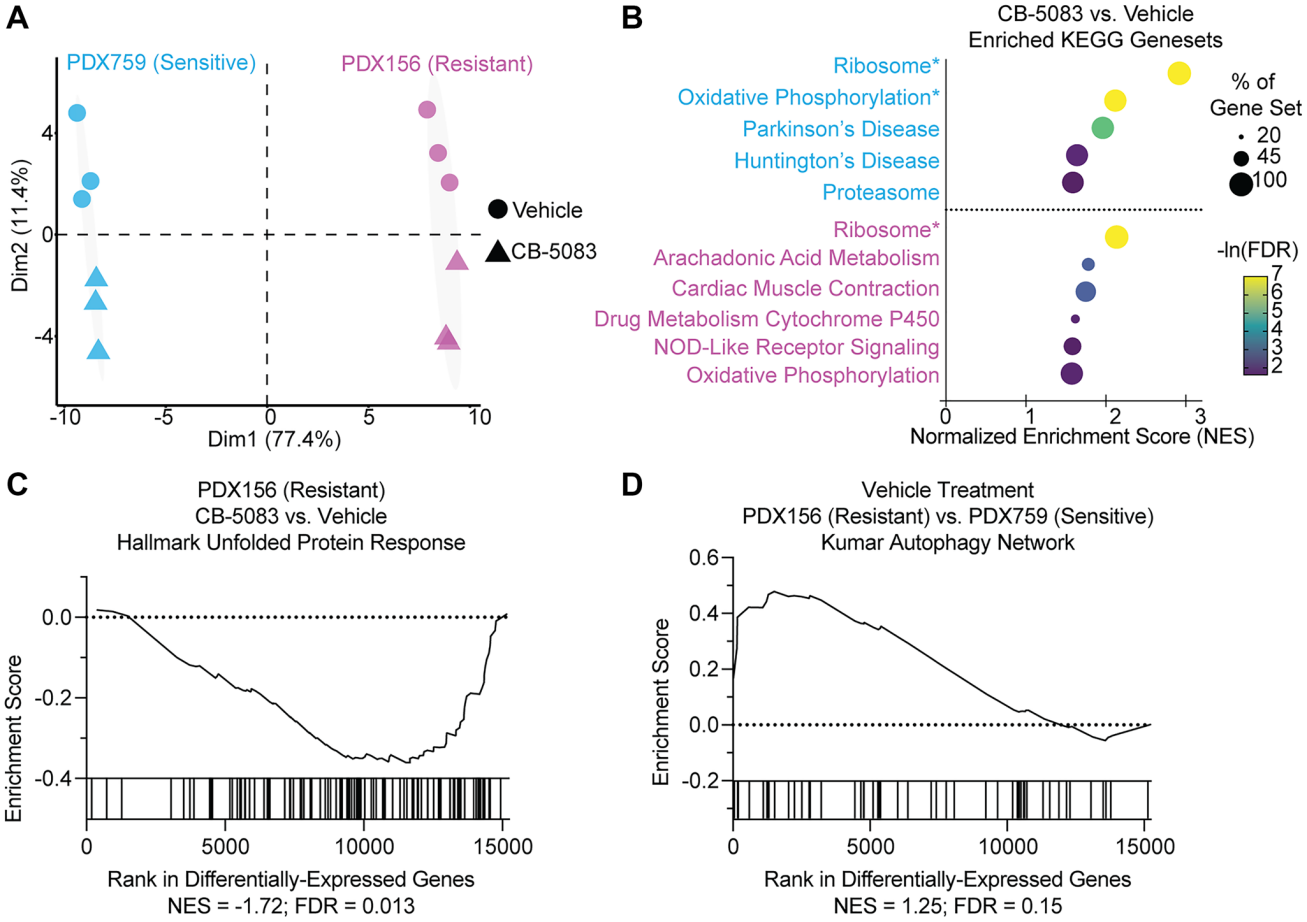
Resistant PDX show decreased UPR signaling and increased autophagy. (**A**) Principle component analysis of whole transcriptome sequencing conducted on the indicated PDX 24 hours after a single dose of either CB-5083 or vehicle. The primary axis of variance hinges on PDX identity rather than treatment, suggesting that drug response is intrinsic to PDX. (**B**) GSEA of differentially expressed genes shows strong enrichment for ribosome and oxidative phosphorylation in the sensitive PDX759, with more modest enrichment in the resistant PDX156. Asterisks indicate gene sets with an FDR of <0.001. (**C**) De-enrichment of the Hallmark UPR gene set in the resistant PDX156 upon CB-5083 treatment compared with vehicle. (**D**) by contrast, PDX156 appears to have a higher autophagy signal basally than the sensitive PDX759.

The data presented above highlight the need to identify biomarkers for the p97-dependent response, an effort which will help guide precision use of proteostasis inhibitors in patient populations [42, 43]. To identify the genes and pathways associated with CB-5083 treatment, we compared transcriptional responses in sensitive (PDX759) and resistant (PDX156) PDX tumors by treating mice with either vehicle or CB-5083 for 24 hours. Tumors were excised and RNAseq was performed in triplicate. Principal component analysis using only differentially expressed genes demonstrated that the first principal component (PC1) accounted for 90% of the variance and was primarily driven by PDX identity (Figure 5A). The second principal component (PC2), which explained 10% of the variance, was attributed to the effect of drug treatment (CB-5083 vs. vehicle).

Although PC2 explained a smaller proportion of the variance, it represented a significant biological effect related to the drug treatment, which we further investigated using gene set enrichment analysis. In fact, CB-5083 treatment was associated with upregulated ribosomal biogenesis in both PDX models, but the effect was more pronounced in the sensitive PDX759 mice (Figure 5B). Oxidative phosphorylation was also higher in the sensitive PDX line. Unresolved ER stress can induce apoptosis through unchecked protein synthesis and production of reactive oxygen species (ROS), potentially linking this transcriptional response to a proteostasis insult [44]. As shown in (Supplementary Figure 3), there was also no enrichment for UPR activation in the sensitive PDX759 model. In contrast, the resistant PDX156 tumors showed downregulation of UPR signaling in the presence of CB-5083 (Figure 5C).

Prior work identified basal and activated levels of autophagy as powerful predictors of resistance to UPR induction and cell death initiated by the HSP70 inhibitor, MAL3-101, in both RMS and breast cancer models [6, 45, 46]. However, as described above, CB-5083 treatment may inhibit autophagy within tumor xenografts since there was a modest increase in the amount of LC3-I, the precursor form of the activated/autophagy-targeted LC3-II species (Figure 3B). We therefore asked whether differential expression of an autophagy signature might further distinguish the sensitive and resistant models. Indeed, gene set enrichment analysis (GSEA) showed elevation of several autophagy markers in the resistant PDX156 model after either vehicle or CB-5083 treatment compared to the sensitive PDX759 model (Figure 5D). To validate this result, we conducted qPCR for the autophagy regulators *ATG3, ATG5,* and *ATG12* in these models and found that CB-5083 induced higher levels *ATG5* and *ATG12* in the resistant PDX156 model, but not in the sensitive PDX759 model (Supplementary Figure 4). Thus, two proteostatic network components—the UPR and autophagy—might be predictive biomarkers of a CB-5083-dependent response in RMS.

### Resistance to MAL3-101 and CB-5083 does not necessarily correlate

**Figure 6:**
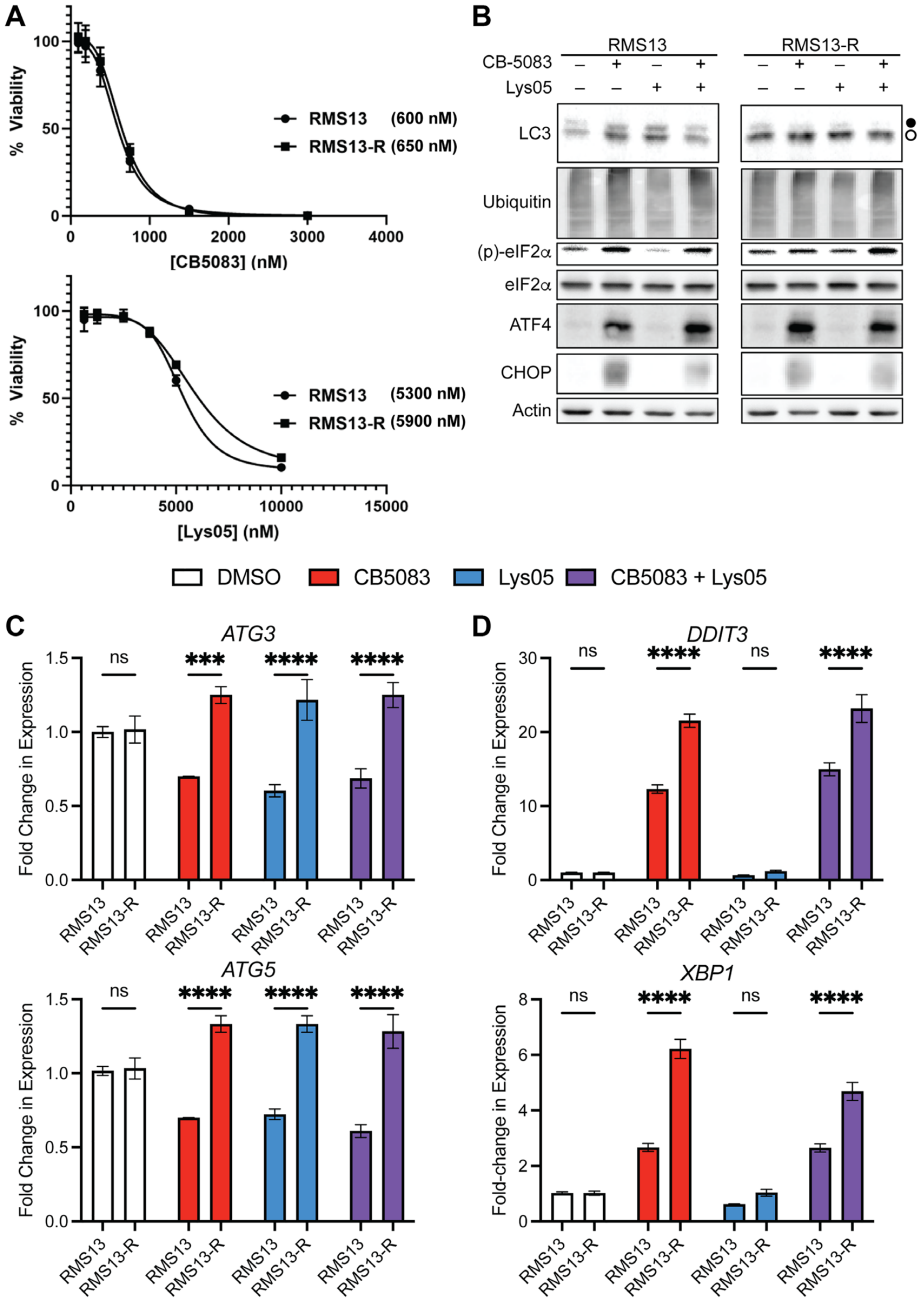
Resistance to proteostasis inhibitors is not cross-reactive, and highlights plasticity. (**A**) A MAL3-101 resistant derivative RMS13 cell line, RMS13-R, was grown through steady dose escalation of the HSP70 inhibitor. This cell line does not exhibit cross-resistance to CB-5083 or Lys-05 as shown through EC_50_ doses, suggesting different upstream mechanisms that converge upon UPR activation. (**B**) CB-5083 is competent to activate the UPR alone and in combination with Lys-05. Closed circle, LC3-I; open circle, LC3-II. (**C**) Levels of autophagy markers *ATG3* and *ATG5* or (**D**) UPR markers *DDIT3* (CHOP) and *XBP1* in RMS13 and RMS13-R cells treated with the indicated drugs. Significance assessed by two-way ANOVA, significant for cell line and interaction but not for drug in *ATG3* and *ATG5*, and significant for cell line, drug, and interaction for *DDIT3* and *XBP1*. Asterisks indicated significance of pairwise comparisons of RMS13 and RMS13-R cells by post-hoc Sidak’s test; ^***^ *p* < 0.001, ^****^ *p* < 0.0001.

Our *in vitro* and *in vivo* data suggest several common features when p97 or HSP70 are inhibited in RMS: the UPR is activated and autophagy activity might be modified (Figures 3B, 5D, and [6]). We therefore asked whether RMS cells activate autophagy to counteract inhibition of the proteostasis network. To do so, we employed a MAL3-101 resistant derivative of the RMS13 cell line that had been isolated through sustained exposure and clonal isolation, RMS13-R [10]. We then tested the sensitivities of RMS13-R and parental RMS13 cells to CB-5083 and Lys05, a dimeric hydroxychloroquine derivative with more potent lysosomal accumulation and inhibitory effects on the autophagy pathway [47]. Surprisingly, the sensitivities of RMS13-R and the parental cell lines to CB-5083 versus Lys05 were nearly identical (Figure 6A). As previously reported [6], the RMS13-R cell line was also characterized by higher basal autophagy (Figure 6B). Interestingly, treatment with either CB-5083, Lys05, or the combination, reduced expression of *ATG3* and *ATG5*, which encode two critical regulators of autophagy in the parental RMS13 cells, but not in the RMS13-R line (Figure 6C). By contrast, both lines showed similar activation of the UPR, as assessed by measuring (p)-eIF2α, ATF4, and CHOP (Figure 6B). The RMS13-R line showed a somewhat heightened transcriptional response of downstream UPR effectors, i.e., CHOP and XBP1 (Figure 6D). Together, we conclude that the cellular mechanisms underlying HSP70 versus CB-5083 resistance are distinct, even though they both activate the same stress response pathway, i.e., the UPR. Future work will be required to define how RMS and cancer cells from different lineages have evolved to withstand the effects of distinct proteostasis damaging drugs, whether there is a causal role of the UPR or other stress response pathways, and which compensatory nodes of the proteostasis network will need to be simultaneously targeted to yield effective therapeutic outcomes.

## DISCUSSION

We previously showed that activation of the pro-apoptotic arm of the UPR, via inhibition of HSP70, provides a novel route to kill RMS cells in culture [6, 10]. Here, we used preclinical models to compare the effects of a specific HSP70 inhibitor versus inhibitors of another central factor in the proteostasis network, p97, with the goal of appraising new therapeutic strategies for patients with RMS and to better understand the determinants required for future clinical applications.

We now show that the HSP70 inhibitor, MAL3-101, only temporarily slowed tumor growth when examined in xenografts, even though the drug effectively killed RMS cells [6, 10]. Since clinically active HSP70 inhibitors are lacking, we used our profiling data to find agents with improved drug-like properties that recapitulate the effects of MAL3-101 and which, ideally, will better control tumor growth. Ultimately, we found that inhibition of p97, via CB-5083 administration, perturbs proteostasis *in vitro* and *in vivo* and significantly slowed tumor growth in a subset of PDX models for RMS.

One major conclusion from our work is the cell context-dependent balance of proteostasis. Although our prior data show that suppression of cytosolic HSP70 activates the UPR in RMS [10], LINCS data strongly connected this transcriptional signature to loss of ER-resident *HSPA5*/GRP78/BiP in other cancer cell lines, including YAPC (pancreatic carcinoma), ES2 (ovarian cancer), AGS (gastric carcinoma), and HT29 (colon cancer). In some cancers, inhibition of this ER lumenal chaperone with tool compounds similarly showed efficacy in several models [48]. Additionally, although *VCP* suppression resulted in UPR activation in both RMS and other cancer contexts, inhibition with CB-5083 resulted in preferential UPR activation in tumor xenografts, rather than in the kidney. While UPR signaling can result in either cellular adaptation or apoptosis, we interpret UPR activation as a biomarker of proteostasis loss in these studies. An RMS-specific enhanced role for *VCP* in safeguarding cell viability may explain why genetic knockdown was difficult to achieve (Supplementary Figure 1). We conclude that the cellular determinants of proteostasis can highlight histology-selective opportunities for therapeutic gain.

The modest single-agent efficacy of CB-5083 *in vivo* may represent either pharmacologic challenges or intrinsic drug resistance in tumors. We hypothesize that both are at play. In support of the former, dose-limiting toxicities required de-escalation of dosing in NSG mice (Figure 3). In favor of the latter, the four models we used demonstrated a clear gradient of intrinsic sensitivity to this strategy. The appearance of autophagy as a hallmark of resistant tumors mirrors our experience with HSP70 inhibition, as well as efforts which more broadly demonstrate a cytoprotective role for autophagy [49–51]. Our data would also suggest, however, that autophagy is insufficient to induce CB-5083 resistance since a cellular model of MAL3-101 resistance was characterized by heightened autophagy but remained CB-5083-sensitive. In addition, combined inhibition of autophagy and p97 failed to enhance UPR induction. Instead, we favor a more nuanced model whereby autophagy may serve as a surrogate marker of a more robust proteostasis network that responds to individual insults, such as HSP70 or p97 inhibition. Future study of these cell line and PDX models may nominate additional pathways or mechanisms of resistance that support or can replace autophagy in triggering CB-5083 resistance. Regardless, the complexity of the proteostasis network may severely limit the effectiveness of any single perturbation in RMS. An alternative approach to enhance the therapeutic effects of CB-5083 is combined targeting of p97 and cytosolic HSP70. MAL3-101 and CB-5083 selectively induce the UPR in RMS cell lines [10] or tumor xenografts (Figure 3C), respectively. Indeed, we previously observed modest synergistic effects when combining MAL3-101 and CB-5083 in the MAL3-101 resistant RMS13-R cell line [6]. However, this effect was absent in the parental line, and was only seen in RMS13-R cells at the highest MAL3-101 doses examined (10 μM). The limited systemic exposure with our current formulation of MAL3-101 (Figure 2B) and the yet-to-be defined toxicities seen at higher doses indicate that it may be challenging to test a MAL3-101 and CB-5083 combination in PDX models. Toxicities seen in patients receiving proteostasis inhibitors are varied. Off-target toxicity (PDE6 inhibition) terminated the clinical development of CB-5083, so on-target effects of p97 inhibition are incompletely described. Proteasome inhibition induces inflammatory toxicities (including pneumonitis) thought to stem from stabilization of NF-kB [52], as well as neuropathy and cardiomyopathies, which are due to mitotoxicity and proteotoxicity, respectively [53–55]. Careful preclinical and clinical study will be needed to define the toxicities of any combination therapy. In addition, efforts to study and improve upon the pharmacokinetics of MAL3-101, and to develop additional HSP70 inhibitors [56, 57], will permit future testing of the hypotheses that HSP70 inhibition improves the CB-5083 response in resistant RMS tumors, and whether this combination can be safely administered.

Patients with high-risk rhabdomyosarcoma continue to suffer poor outcomes despite clinical trials of intensified cytotoxic chemotherapy and radiation [1]. The data presented here suggest that as newer agents are developed—with an eye towards precise disruption of proteostasis networks—preclinical testing in RMS should be a priority. Furthermore, the development of autophagy as a biomarker of intrinsic resistance to these strategies, and/or biomarkers of UPR signaling as a measure of target engagement, can also now be pursued in PDX models to predict heterogeneous responses.

Finally, as a result of this work, we hypothesize that any single proteostasis inhibitor will be insufficient to exploit the RMS vulnerabilities we have described. Instead, predicting and disabling compensatory responses within the proteostasis network to any single inhibitor will be essential to guide the clinical development of proteostasis-targeted therapies. Given the propensity of relapsed RMS to transiently respond to chemotherapy, combinations of genotoxic and proteotoxic therapies may yield an alternative means to improve the impact of proteostasis inhibitors. This goal will be pursued in the future.

## MATERIALS AND METHODS

### Cell culture

Cell lines were obtained from the Children’s Oncology Group COGCell Repository (Rh30, Rh41) or purchased from ATCC (RMS13, RD) or Takara Bio (Lenti-X). RD and Lenti-X cells were grown in DMEM, and the remaining lines were grown in RPMI-1640, both supplemented with 10% FBS and 1x penicillin/streptomycin, in a 37° C incubator with 5% CO_2_. Cells were tested quarterly for mycoplasma and tested to confirm identity by STR analysis twice a year.

### Immunoblots

Cells for immunoblots were lysed in ice cold RIPA buffer with protease and phosphatase inhibitors (Roche). Lysates were quantified using a DC protein assay (Bio-Rad), boiled in 1x Laemmli buffer, and then run on a 4-16% TGX gel. Gels were transferred onto nitrocellulose membrane and blotted overnight in primary antibody, washed three times, and then incubated with HRP-conjugated secondary antibody for one hour. Blots were washed twice, then imaged using ECL reagent (Amersham) on a GelDoc (Bio-Rad). Blots are representative of at least three independent replicates. Quantitation of band intensities was carried out by ImageJ.

### Lentiviral transduction and shRNA knockdown

Third generation lentiviral plasmids containing shRNA targeting VCP were purchased from Sigma. To generate lentiviral particles, Lenti-X cells were transfected with plasmids of interest, pCMVdR8.91, and pMD2.g using TransIT-LT1 transfection reagent (Mirus) at a 3:1 ratio. Six hours later, ViralBoost reagent (Alstem) was added at 1:500. Seventy-two hours after transfection, viral particles were harvested from the supernatant, filtered through a 0.45 micron PES syringe, and then added to target cells with 6 mg/mL polybrene. In twenty-four hours, cells were plated in puromycin for three days.

### Antibodies

Antibodies from the following sources were used for immunoblotting. Cell Signaling Technologies: PERK (#5683), ATF4 (#11815), CHOP (#2895), eIF2α, (#5324), phospho-eIF2α (#3398), LC3 (#4108), ubiquitin (#20326), HRP-conjugated anti-mouse (#7076), HRP-conjugated anti-rabbit (#7074). ProteinTech: CHOP (15204-1-AP); Actin (66009-1-Ig).

### RNA extraction and quantitative PCR

Total RNA was extracted from cells or tissues using an RNEasy kit (Qiagen). For quantitative PCR, cDNA was synthesized using a SensiFast cDNA kit (Bioline) using manufacturer’s instructions from 250 ng of RNA. The cDNA was diluted 1:4, and 8 uL was added to 400 nm forward and reverse primers (shown in Supplementary Table 1) and 2× Fast SYBR Green mastermix (Thermo). Thermocycler settings were 95ºC × 10 minutes to denature, followed by 30 cycles of 95ºC × 30 seconds, 60ºC for 90 seconds, and 72ºC for 45 seconds. Relative expression was calculated using the ΔΔCT method, normalizing to GAPDH.

### RNA Sequencing

Libraries from xenografts (conducted in triplicates) were prepared from extracted RNA using Stranded mRNA Prep kits (Illumina), validated on the Agilent TapeStation (Agilent Technologies, Palo Alto, CA, USA), and quantified by using Qubit 2.0 Fluorometer (Invitrogen, Carlsbad, CA, USA). Sequencing of RMS cell lines in duplicates was previously described [10] and reads downloaded from GEO (accession GSE80525). Processed reads were mapped to the human reference genome (hg38) using the STAR aligner (v2.5.1b). Gene-level expression was quantified using STAR’s ‘quantMode’ feature with GENCODE p5 annotations. Quality control metrics were evaluated using ngsutilsj (v0.3-2180ca6). Subsequent analyses were performed in R (v3.5.3). Aligned reads were normalized using the trimmed mean of M-values (TMM) method (EdgeR v3.24.3), and counts were log2(cpm + 1) transformed. The VOOM function (limma package) was used to estimate the mean-variance relationship and assign precision weights, which were then integrated into the empirical Bayes linear modeling framework (limma v3.38.3) to calculate statistical outputs, including *p*-values, adjusted *p*-values, and log-fold changes (LogFC). Pathway analysis was conducted with Gene Set Enrichment Analysis [58]. Differentially expressed genes with an absolute LogFC of ≥1 and a *p*-value of ≤0.05 were analyzed by SigCom LINCS [21]. RNASeq data generated in this study are available in Gene Expression Omnibus (GEO), record GSE270783.

### Cell line xenograft experiments

RMS13 cells trypsinized, washed in PBS, and suspended in 50% Matri-Gel (Corning), then injected subcutaneously into the flanks of *nu/nu* mice (UCSF Helen Diller Family Comprehensive Cancer Center colony). Animals were monitored twice weekly and diameters of subcutaneous tumors were measured by calipers. Animals with a body condition score of <3 or weight loss >20% were euthanized. After 21 days of treatment, all mice were humanely euthanized following IACUC protocols.

### Patient-derived xenograft (PDX) experiments

PDX were established through an IRB-approved protocol at UCSF, or obtained through the Childhood Solid Tumor Network (St. Jude Children’s Research Hospital) [59]. Viably frozen PDX were thawed, washed with PBS, resuspended in 50% MatriGel (Corning) and implanted in the flanks of NSG mice. Animals were euthanized when tumors reached 2 cm in maximum dimension following IACUC protocols, and tumors were surgically extracted, macerated with a scalpel, and digested in buffer containing 0.1% collagenase (Sigma), 0.1% BSA, 20 mM HEPES, 1 μM CaCl_2_, 1.25% Kolliphor-P188 (Sigma), and DNAse for one hour. Cells were strained, washed thrice, and 1 million viable cells in 50% MatriGel were implanted subcutaneously into the flanks of recipient mice for therapeutic studies. Animals were monitored daily, and tumor diameters measured twice a week to calculate volumes. For pharmacodynamic studies, animals were euthanized 24 hours after drug treatment, and implanted tumors were extracted with sterile scissors and flash frozen in liquid nitrogen prior to grinding and resuspension in RIPA (for immunoblots) or buffer RLT (Qiagen) supplemented with beta-mercaptoethanol (for RNA analysis).

### Pharmacokinetic measurements

At pre-specified timepoints, animals were humanely euthanized using CO_2_ inhalation and bilateral thoracotomy. Blood was obtained through cardiac puncture and spun in EDTA tubes at 1000 g for 10 minutes, and plasma was isolated and flash frozen in cryovials immersed in liquid nitrogen. Samples were shipped to Integrated Analytical Solutions (Berkeley, CA, USA). Quantitative LC/MS-MS assays and calibration standards were developed using serial dilutions of MAL3-101 and CB-5083 in mouse plasma.

### Chemical compounds

MAL3-101 was generated as described [16, 18]. CB-5083 was either a gift from Cleave Biosciences or purchased from Selleck Chemicals. UPCDC-30766 was synthesized as described [36]. Lys05 was purchased from Selleck Chemicals. For *in vitro* experiments, MAL3-101 and CB-5083 were dissolved in DMSO and Lys05 was dissolved in PBS. For *in vivo* experiments, MAL3-101 was dissolved in 25% Kolliphor-HS (Sigma), 15% ethanol, and 10% dimethylacetamide (Sigma) and delivered by intraperitoneal injection. CB-5083 was suspended in sterile 0.5% methylcellulose using a mortar and pestle and administered by oral gavage. Lys05 was resuspended in sterile PBS and administered by intraperitoneal injection.

## SUPPLEMENTARY MATERIALS



## Abbreviations

ERAD: Endoplasmic reticulum-associated degradation
GSEA: gene set enrichment analysis
MTD: maximum tolerated dose
PDX: patient-derived xenograft
RMS: rhabdomyosarcoma
UPR: unfolded protein response
