# Supplementary material for: In vivo manipulation of the protein homeostasis network in rhabdomyosarcoma
Source: Oncotarget. 2025 Aug 29;16:681–96. doi: 10.18632/oncotarget.28764 (PMC12581410; doi:10.18632/oncotarget.28764)
Supplement: Supplementary file 1 [file oncotarget-16-28764-s001.pdf]

In vivo manipulation of the protein homeostasis network in rhabdomyosarcoma

SUPPLEMENTARY MATERIALS

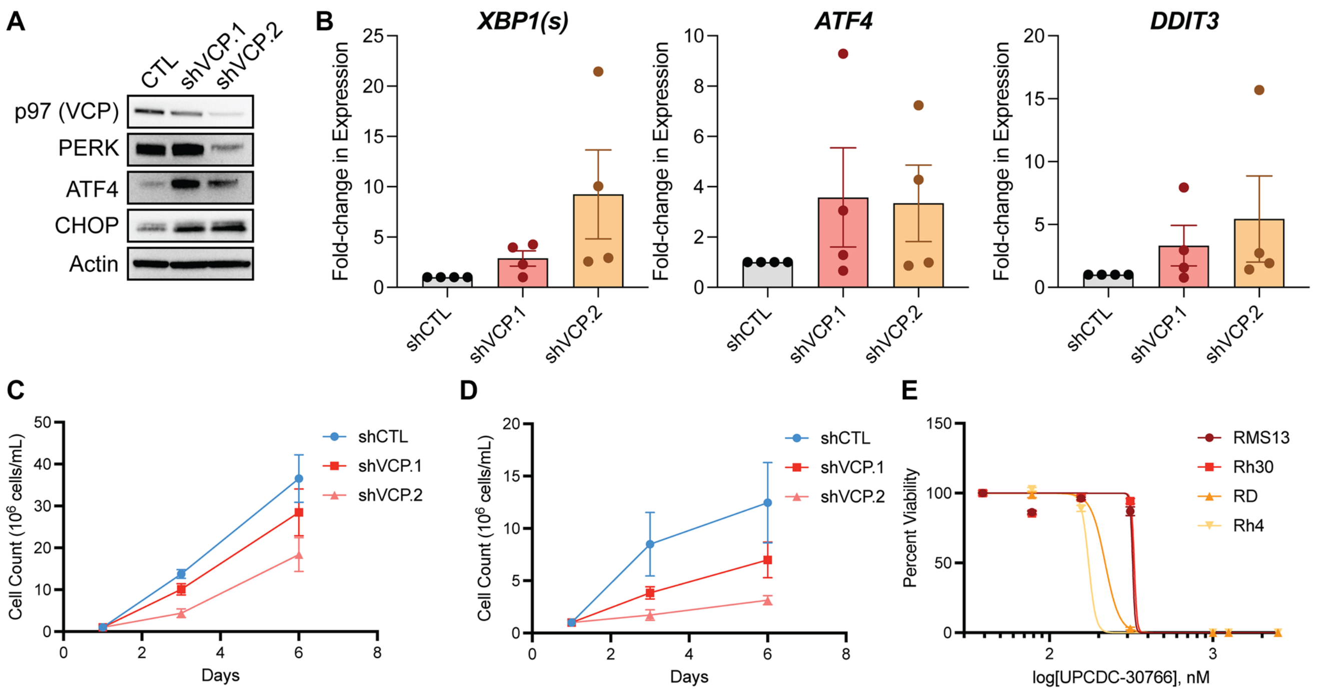

Supplementary Figure 1: Knockdown of VCP activates the UPR and decreases viability in RMS cells. RMS13 cells were transduced with the indicated shRNAs targeting *VCP* or control and selected with puromycin for three days. (**A**) immunoblot showing degree of knockdown and induction of ATF4 and CHOP as markers of UPR activation. Phosphorylated, active PERK demonstrates a slower migration and upward shift. (**B**) quantitative PCR shows a trend towards induction of alternatively spliced *XBP1, ATF4, and DDIT3* (*p-value*s non-significant by one-way ANOVA). Bars show mean +/− SEM. RMS13 (**C**) and Rh30 (**D**) cells were transduced with the indicated shRNA, selected with puromycin for 72 hours, then seeded in equal numbers and grown in continuous puromycin. Cells were counted on days 1, 3, and 6 in three independent replicates. Error bars show standard error of the mean. E, the indicated cell lines were treated with UPCDC-30766 for 6 days, and viability was measured by Alamar Blue.

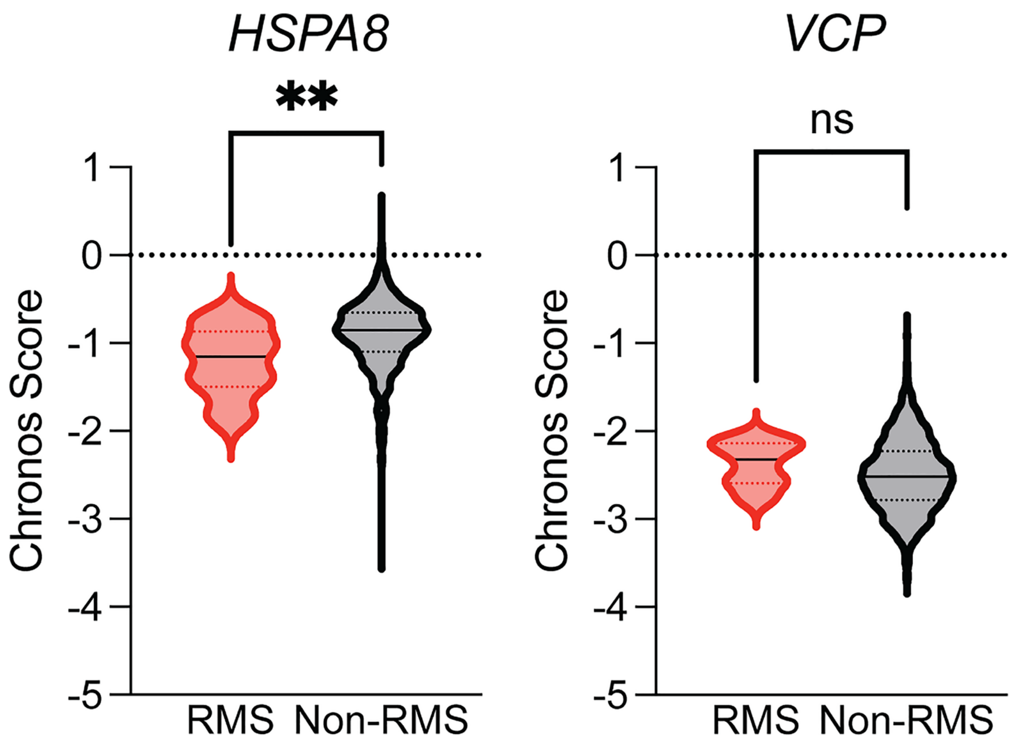

Supplementary Figure 2: Genetic dependencies on proteostasis nodes. Chronos dependency scores from the Dependency Map for *HSPA8* (targeted by MAL3-101) and *VCP* (targeted by CB-5083). Dependency scores in RMS cell lines were compared to all other cell lines using a Mann-Whitney test; $^{**}p < 0.01$; Abbreviation: ns: non-significant.

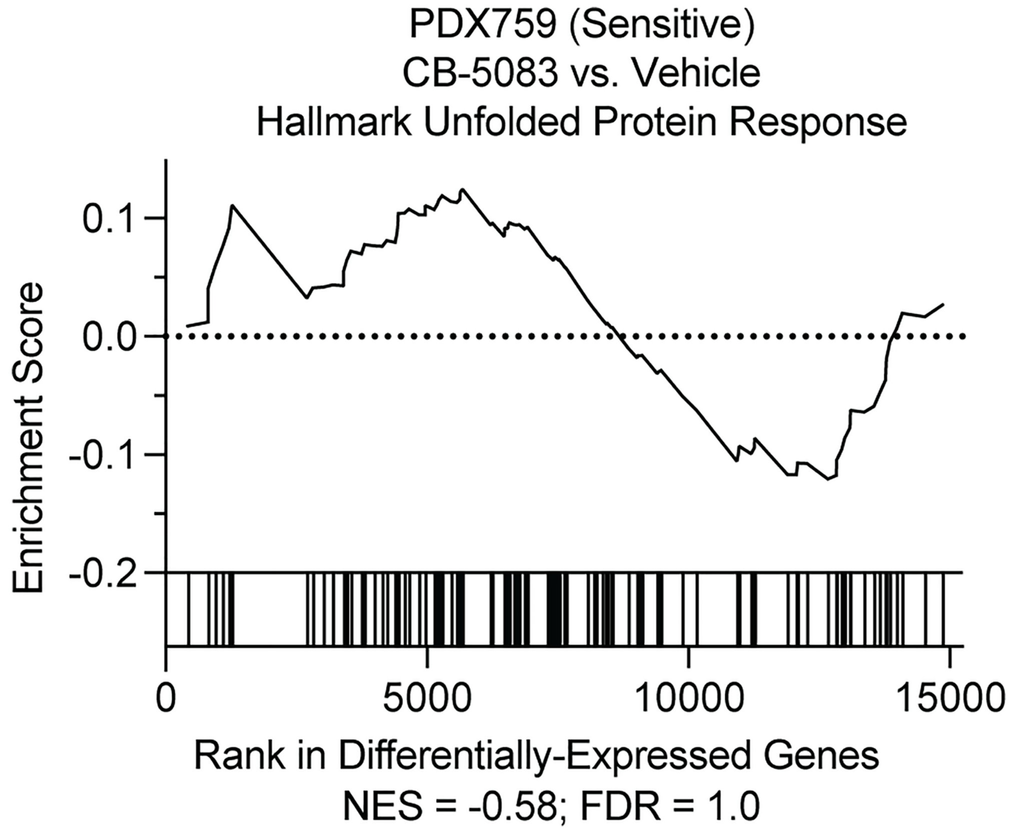

Supplementary Figure 3: Lack of UPR transcriptional signature in sensitive PDX. The Hallmark UPR gene set showed no enrichment in RNASeq of PDX759 treated with CB-5083 versus vehicle.

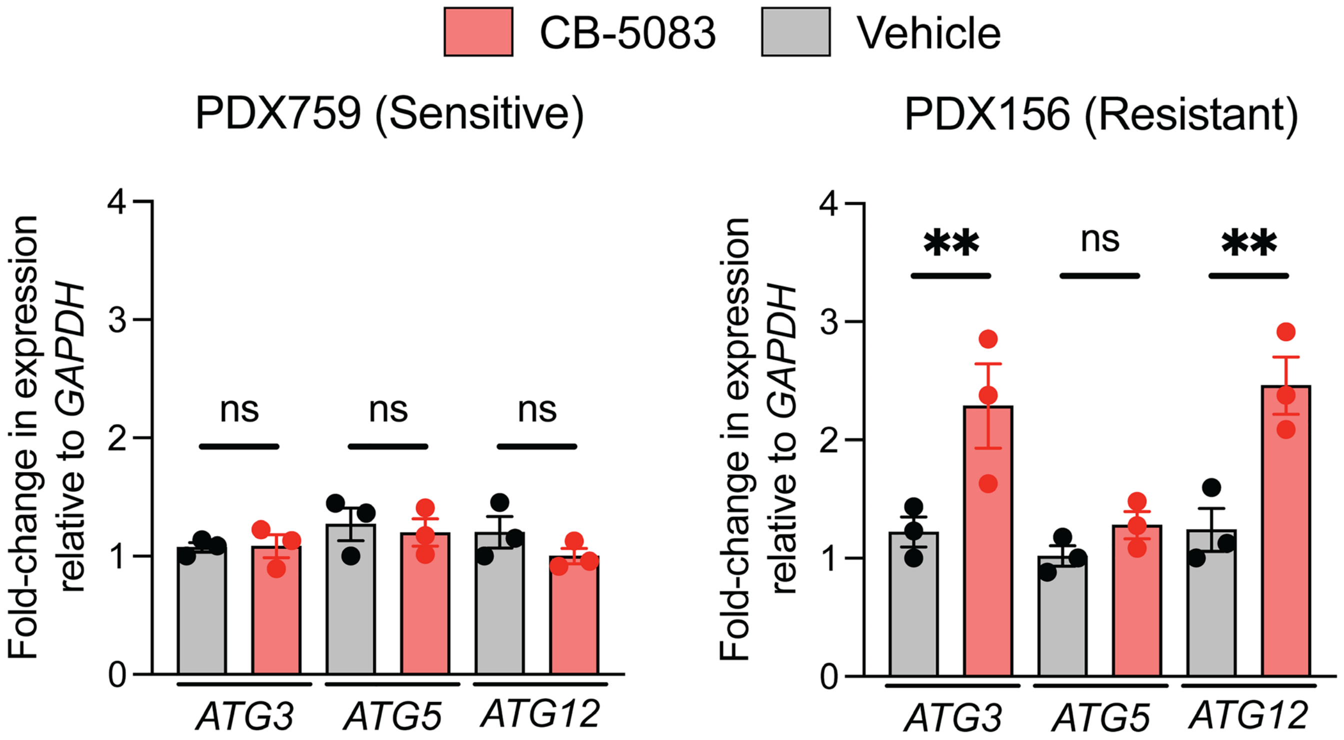

Supplementary Figure 4: Autophagy induction in resistant PDX. Quantitative PCR for *ATG3, ATG5*, and *ATG12* from PDX extracted from mice treated with either vehicle or CB-5083, as in Figure 5. Differences were analyzed by one-way ANOVA followed by a post-hoc Sidak's test. Abbreviation: ns: not significant; ** adjusted p-value <0.01.

Supplementary Table 1: Quantitative PCR primers used

Primer	Sequence
ATF4 forward	ATGACCGAAATGAGCTTCCTG
ATF4 reverse	GCTGGAGAACCCATGAGGT
ATG3 forward	ACATGGCAATGGGCTACAGG
ATG3 reverse	CTGTTTGCACCGCTTATAGCA
ATG5 forward	AAAGATGTGCTTCGAGATGTGT
ATG5 reverse	CACTTTGTCAGTTACCAACGTCA
DDIT3 forward	TTAAGTCTAAGGCACTGAGCGTATC
DDIT3 reverse	TGCTTTCAGGTGTGGTGATG
GAPDH forward	TGCACCACCAACTGCTTAGC
GAPDH reverse	GGCATGGACTGTGGTCATGAG
XBP1(s) forward	AAACAGAGTAGCAGCTCAGACTGC
XBP1(s) reverse	TCCTTCTGGGTAGACCTCTGGGAG
